# Parenting-Related Social Networking Site Use and Psychological Distress in Parents of Infants: Cross-sectional Study Exploring the Moderating Effects of Loneliness and Parenting Anxiety

**DOI:** 10.2196/59029

**Published:** 2024-10-11

**Authors:** Ryuta Onishi

**Affiliations:** 1 Faculty of Nursing, Toyama Prefectural University, Toyama City, Toyama Prefecture, Japan

**Keywords:** social networking sites, social media, psychological distress, loneliness, anxiety, social support, mother, father, infant, psychological, distress, children, web-based questionnaire, parent

## Abstract

**Background:**

In the digital age, social networking sites (SNSs) have revolutionized the approach to parenting. These platforms, widely used to access parenting information and support, affect parents both positively and negatively, with negative effects potentially increasing for those experiencing loneliness or anxiety.

**Objective:**

This study examined the relationship between SNS use and psychological distress among parents of young children, controlling for the moderating effects of loneliness and parenting anxiety. We hypothesized that higher SNS use correlates to greater psychological distress, particularly among parents with elevated levels of loneliness or parenting anxiety.

**Methods:**

A cross-sectional survey design using a closed web-based questionnaire was employed. Participants included 429 parents (205 mothers and 224 fathers) of children aged 0‐3 years recruited through a web-based survey company in Japan. The majority of the participants were couples, with some living with extended family members. The sample also encompassed individuals in cohabiting partnerships and single parents. The survey included measures of psychological distress, loneliness, parenting anxiety, frequency of SNS use for parenting, and covariates. Analytical models to explain psychological distress included interactions between loneliness or parenting anxiety and SNS use, individually for both fathers and mothers.

**Results:**

For mothers, a significant interaction effect was determined only between parenting anxiety and SNS use (*b*=0.247, SE 0.091; *P*=.008). Meanwhile, for fathers, significant interaction effects were observed for both loneliness (*b*=0.324, SE 0.127; *P*=.012) and parenting anxiety (*b*=0.144, SE 0.069; *P*=.038) with SNS use. A simple slope analysis for mothers indicated that SNS use was related to psychological distress only at higher levels of parenting anxiety (*b*=0.304, SE 0.090, *β*=.317; *P*<.001). Among fathers, SNS use was associated with psychological distress at higher levels of either parenting anxiety (*b*=0.330, SE 0.069, *β*=.346; *P*<.001) or loneliness (*b*=0.390, SE 0.098, *β*=.409; *P*<.001).

**Conclusions:**

The study concluded that the relationship between SNS use and psychological distress among parents of young children is moderated by loneliness and parenting anxiety. The findings highlight the need for tailored approaches to help parents manage SNS use, particularly focusing on those with higher levels of loneliness and parenting anxiety. It is imperative that health professionals provide nuanced guidance to parents on SNS use, considering individual psychological factors and potential gender differences in the impact of SNSs on mental well-being.

## Introduction

In the wake of rapid digitalization, social networking sites (SNSs) such as Facebook, X (formerly Twitter), and Instagram have changed the landscape of parenting. Parenting-related SNS use is increasingly common, with parents employing these platforms to obtain parenting information and support [1,2]. This specific use of SNSs in parenting has both positive and negative implications. Parenting-related SNS use provides access to a wide range of information that may be unavailable offline [3,4] and offers solutions related to parenting, particularly by expanding maternal social networks [5,6]. However, parenting-related SNS use tends to disseminate idealized images of parenting, posing a risk of loss of confidence due to unrealistic expectations and pressures [7]. The idealized parenting images prevalent on SNSs may prompt parents to compare their parenting methods with others’, potentially triggering depressive symptoms and negative emotions [8,9]. Given the dual nature of parenting-related SNS use, it is critical to determine methods of use that minimize drawbacks while maximizing benefits.

Among the negative impacts of social media use is psychological distress, which is generally characterized by emotional experience marked by symptoms of depression and anxiety [10]. It has been theoretically demonstrated that psychological distress can impair parenting practices [11], and understanding its mechanisms is crucial for supporting effective parenting functions. The impact of social media use on mental health varies widely [12], highlighting the need to focus on the differences in effects based on users’ backgrounds when exploring the relationship between psychological distress and social media.

Psychological distress associated with SNS use may be driven by the loneliness experienced by parents. Loneliness can be defined as a “negative psychological state in which there is a discrepancy between one’s actual and desired social relationships” [13]. Parental loneliness raises the risk of child maltreatment and may be associated with potential maladjustment in children [14,15]. Lonely people tend to prefer social interactions on the web, reducing the time spent undertaking offline interactions [16]. Although they use social media to compensate for the lack of offline interpersonal relationships, they may not always realize satisfying web-based social relationships [17,18]. Consequently, parents with higher levels of loneliness may rely on limited social relationships through SNSs and experience greater psychological distress owing to unsatisfactory connections. Moreover, the correlation between mothers’ high levels of loneliness and the negative emotions experienced through social comparisons on SNSs [19] suggests that the degree of loneliness may influence psychological distress on SNSs.

In addition to loneliness, parenting anxiety may influence parents’ psychological distress driven by SNS use. Parenting anxiety, characterized by confusion and fear regarding a child’s health, development, and parenting methods, could increase the risk of child maltreatment and child anxiety disorders [20,21]. Parents with high levels of parenting anxiety reportedly use social media more frequently and consider it useful in alleviating anxiety [22]. However, in general, people tend to share information focusing on the successful aspects of their lives on SNSs, facilitating upward comparisons with others who appear to have achieved their goals [23]. Numerous studies have reported that such upward comparisons on SNSs could have negative effects on mental health [24-26]. Given the prevalence of idealized images of parenting on SNSs [7], parents with high parenting anxiety may be increasingly vulnerable to psychological distress from upward comparisons. In addition, elevated parenting anxiety often correlates with lower parenting self-efficacy and self-esteem [27]. Lack of parenting self-efficacy may attenuate the comforting and anxiety-reducing effects of addressing concerns through smart devices [28].

Systematic reviews of the relationship between SNSs, loneliness, and social anxiety indicate that individuals who are lonely or experience social anxiety are more likely to engage in problematic social media use [29], suggesting that such individuals may be increasingly vulnerable to psychological distress from SNSs. Despite the accumulation of knowledge on the relationship between social media, SNSs, loneliness, anxiety, and depression, research in the context of parenting remains limited [30]. Knowledge regarding SNS use among parents of young children is particularly limited, and studies concerning social media use in parenting have predominantly focused on mothers. Exploring the relationship between SNS use and psychological distress in parenting, taking into account levels of loneliness and parenting anxiety for both fathers and mothers, may provide nuanced evidence for SNS use in parenting and professional support.

The usage of SNSs exhibits cultural differences [31], and Japan has its own unique cultural characteristics. In Japan, the widely known international platforms, such as X (formerly Twitter), Instagram, and Facebook, are used alongside LINE, which is popular in some parts of Asia [32]. Compared with other countries, Japanese users tend to employ SNSs more for browsing than for sharing information [33]. SNS use comprises 2 aspects: active and passive use [34], with a tendency for passive use to be more prevalent in Japan. Passive use of SNSs is often linked to negative impacts on mental health [35]. As Japanese users are predicted to be a group potentially at risk of psychological stress due to SNS use, it is significant to focus on this issue.

This study elucidates the moderating effects of loneliness and parenting anxiety on the relationship between SNS use and psychological distress among mothers and fathers of young children. This study focuses on the individual experiences of fathers and mothers, as it is predicted that their parenting roles differ qualitatively owing to sociocultural and biological factors, despite sharing commonalities as parents. In Japan, there is a strong gender role division where fathers are seen as responsible for work outside the home, while mothers are responsible for household chores and child-rearing duties [36]. As of 2021, surveys on parenting time in Japan reveal a significant disparity in the time spent on child rearing, with wives contributing substantially more time than husbands [37].

**Figure 1.**
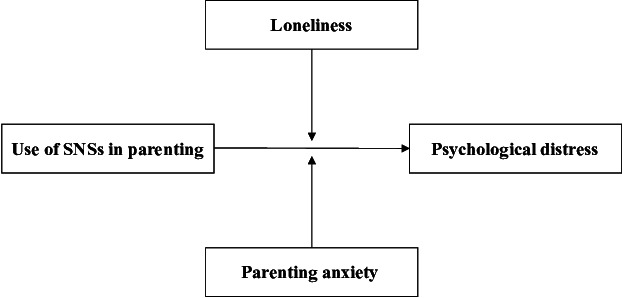
Hypothetical conceptual model. SNS: social networking site.

In addition, it is generally observed that women engage in the exchange of support on social media more frequently than men [38]. While it is widely recognized that mothers use social media to share information and support regarding child-rearing [5], social media platforms specifically targeting fathers are limited [39], indicating a gender disparity in web-based parenting resources. Therefore, it is crucial to conduct analyses that consider gender differences. Moreover, lonely individuals may lack a sense of fulfillment from social relationships on social media [17,18]. Upward comparisons, which are more likely to occur on these platforms, may particularly harm the mental health of individuals with parenting anxieties [24-26]. These risks can affect both fathers and mothers. Accordingly, we constructed a hypothetical conceptual model (Figure 1), and the following hypotheses were formulated:

- Hypothesis 1: Mothers use SNSs for parenting more frequently than fathers.
- Hypothesis 2: Only when mothers and fathers have high levels of parenting anxiety, frequent SNS use for parenting is associated with high psychological distress.
- Hypothesis 3: Only when mothers and fathers have high levels of loneliness, frequent SNS use for parenting is associated with high psychological distress.

## Methods

### Study Design

This study used a cross-sectional design with a closed web-based questionnaire. To ensure the quality and transparency of research, the work employed the Survey Reporting Checklist (CROSS) developed by Sharma et al [40] and the CHERRIES (Checklist for Reporting Results of Internet E-Surveys) developed by Eysenbach [41]. This study reports a subset of data from the “Information Behavior on Social Networking Services Among Parents Raising Infants” survey.

### Operational Definitions

In this study, SNSs were defined as communication platforms where participants can consume, produce, and interact with a stream of content generated by connections on the site [42]. Specific SNSs considered were X (formerly Twitter), Instagram, Facebook, and YouTube, taking into account the context of Japan. LINE, which is widely known in Japan, was excluded from this category owing to its limited use of the feed function characteristic of SNSs and its primary use as a tool for routine communication with acquaintances. The use of SNSs for parenting was operationally defined as usage to gather information related to parenting, monitoring other families, and building social relationships.

### Participants

This study included 429 parents of 0- to 3-year-old children. The eligibility criteria required that the participants were actively parenting at least 1 child aged between 0 months and 3 years and 11 months at the time of the survey. There were no restrictions based on the health status of the parents or children or on family structure. The exclusion criterion was the absence of any experience using SNSs for parenting-related activities.

Sampling was conducted using convenience sampling from the national panel managed by Cross Marketing, Inc, a web-based survey company in Japan, which manages more than 5 million active web users and provides academic web-based survey services to a wide range of demographic groups. The company’s active panel comprises a wide variety of web users, creating a large-scale monitor that is not biased by influx from specific sites or advertisements. The recruitment process began with a one-time survey invitation sent by the survey company to a randomly selected group of approximately 2000 individuals deemed eligible for this study. Those who received the invitation first answered a question regarding their child’s age; inconsistent responses were excluded. Subsequently, individuals who agreed to participate in the study were directed to the web-based questionnaire link via a click of an “agree” button after reading the research request document prepared by the authors. The study aimed to include 200 fathers and 200 mothers, and distribution continued randomly until this goal was reached, at which point the survey distribution was halted.

### Data Collection

Data were collected in August 2023 using a web-based survey. The questionnaire was developed and administered by the authors using the Qualtrics web-based survey system [43]. Qualtrics ensures data management through encryption, redundancy, continuous monitoring, and single sign-on, and is FedRAMP-authorized as well as ISO27001-certified. The questionnaire was designed by the authors to be user-friendly and was pretested to avoid design issues. Participants who completed the questionnaire received a web-based reward based on Cross Marketing, Inc standards.

### Measures

The survey included sections on SNS use, SNS use for parenting, psychosocial characteristics, and basic demographics. The questionnaire was developed through the following process. First, the content validity of the questionnaire was reviewed by a researcher (RO) with extensive experience in public health nursing practice and research, who also has experience in child-rearing support. Potential common errors in the questionnaire (eg, double-barreled questions, confusion, and leading questions) were checked by a representative from Cross Marketing Inc, who has extensive experience in designing web-based surveys, and appropriate revisions were made. In addition, face validity for all survey items was ensured through pretesting with 4 university students and 5 mothers with infants and older children. Owing to the limitations of the pretest sample size, the factor structure and internal consistency were confirmed in the main survey. Details of the survey items are shown in Multimedia Appendix 1.

### SNS Use

Data were collected with regard to the possession of digital devices (smartphones, tablets), currently used SNSs (X, Instagram, Facebook, YouTube, LinkedIn, etc), and overall frequency of SNS usage by participants. The frequency of SNS usage, based on previous research [34], considered both active and passive usage aspects. Active usage was mainly related to information production (eg, posting updates on SNSs, sending private messages), while passive usage concerned information consumption (eg, scrolling through news feeds and viewing other users’ profiles). The frequency of active and passive SNS usage over the past week was assessed, with response options ranging from 1 (not at all) to 8 (more than 10 times a day).

### SNS Use for Parenting

Considering the lack of a specific scale to measure SNS use for parenting, original measurement items were created. Based on previous studies [44] and interview data from mothers in the parenting phase, 3 categories were formulated for SNS use in parenting: “information gathering,” “monitoring,” and “connecting.” Each category consisted of 2 items, with “information gathering” including items related to gathering information on parenting and children. “Monitoring” included tracking other families’ parenting and their children’s conditions, while “connection” included making friends in parenting and communicating with peers in parenting. Items were measured on a 5-point scale (1=never use to 5=always use), with the mean value calculated within a range of 1‐5, and higher scores indicating greater subjective frequency of use.

An exploratory factor analysis using maximum likelihood promax rotation was conducted to determine the handling of the scale for SNS use in parenting. The applicability of factor analysis was confirmed by ensuring that the Kaiser-Meyer-Olkin measure was greater than 0.8, and Barlett’s test of sphericity was significant. The number of factors was determined by examining the scree plot and using eigenvalues greater than 1. A factor loading of 4.0 or higher was employed as the criterion for item selection. Reliability was assessed by calculating Cronbach α.

Table 1 shows the results of the exploratory factor analysis. Bartlett’s sphericity test for the 6 items measuring SNS use in parenting was significant (*χ*²_15_=2319.43; *P*<.001), and Kaiser-Meyer-Olkin values were above 0.8, confirming the suitability of the data for exploratory factor analysis. Exploratory factor analysis with maximum likelihood promax rotation revealed a 1-factor structure for the 6 items (Table 1). An attempt was made to confirm a 3-factor structure considering the 3 categories, but eigenvalues for the factors after the first were lower than 1, indicating that the model was not suitable. Therefore, the study measured SNS use for parenting by employing the total score of all items, with a Cronbach α value of 0.92.

**Table 1.** Exploratory factor analysis (N=429).

Statements	Factor loading	Communality
Parenting insights browsing	0.93	0.86
Observing childhood moments	0.91	0.82
Gathering parenting-related information	0.80	0.65
Gathering child-related information	0.78	0.61
Building a parenting community	0.72	0.52
Communication among parenting peers	0.72	0.52

### Psychosocial Factor

#### Psychological Distress

The Japanese version of the K10 scale developed by Kessler et al [45,46], a globally recognized gold standard screening tool for mood and anxiety disorders in the general population, was used to measure the level of psychological distress but not in screening for mental disorders; therefore, no cutoff value was applied. Higher scores indicated greater levels of psychological distress. Sample items were rated on a 5-point Likert scale (1=not at all to 5=all the time), and the mean was calculated within a range of 1‐5. Cronbach α value for this study was 0.95.

#### Parenting Anxiety

A subscale of the Parenting Emotions Scale developed in Japan [47] was employed to measure parenting anxiety. The parenting anxiety subscale, one of the negative emotion subscales, includes aspects of anxiety about the child’s development and parenting methods, with established reliability and validity [47]. For example, items such as “Feeling unsure about how to parent” were rated on a 4-point Likert scale (1=not at all to 4=often), with the mean value calculated within a range of 1‐4, and higher scores indicating greater parenting anxiety. Cronbach α value for this study was 0.86.

#### Loneliness

The Japanese version of the 10-item short form of the UCLA Loneliness Scale (Version 3) [48,49], validated among mothers of infants, was employed. Items were rated on a 4-point Likert scale (1=not at all to 4=always), with the mean value calculated within a range of 1‐4, and higher scores indicating greater feelings of loneliness. Cronbach α value for this study was 0.79.

#### Social Support

A subscale related to support from significant others from the Japanese version of the Multidimensional Scale of Perceived Social Support [50-53] was also used. It includes 4 items rated on a 7-point Likert scale (1=very strongly disagree to 7=very strongly agree), with higher scores indicating higher perceived social support. Example items include “I have someone with whom I can share joys and sorrows.” The average score for each subscale was calculated on a scale of 1‐7. The reliability and validity of this scale have been previously established [50]. Cronbach α value for this study was 0.91.

### Participant Characteristics

Data were collected on participants’ age, number of children, age of youngest child, cohabiting family members, occupation, level of education, subjective economic status, and division of childcare and housework.

### Statistics

Initially, descriptive statistics were first applied for all variables in the study, stratified by fathers and mothers. To identify differences between variables for fathers and mothers, *t* tests or chi-square tests were performed. Thereafter, separate multiple regression models were constructed for mothers and fathers, with psychological distress as the outcome variable and SNS use for parenting, parenting anxiety, and loneliness as predictor variables. Interaction terms between SNS use for parenting and parenting anxiety and loneliness were included in the model to examine their interactions. Covariates included parental age, which influences affinity for digital devices; number of children and age of youngest child, which influence childcare realities; subjective economic status, which was found to be related in univariate analyses; and active or passive use, reflecting the impact of SNS use in contexts other than parenting. Comprehensive checks were undertaken to ensure that the necessary assumptions for the use of multiple regression analysis were met. First, the skewness and kurtosis of the dependent variable were evaluated to ensure that the analysis was not affected by nonnormality. Second, the linear relationship between the dependent and independent variables was visually assessed using scatter plots. Third, the Durbin-Watson ratio was confirmed to be around 2 to ensure the independence of the observations. Fourth, normality and homoscedasticity of errors were visually assessed using normal Q-Q plots of residuals and studentized residuals. Fifth, multicollinearity was assessed by ensuring that the variance inflation factor for independent variables was lower than 10. Moreover, for significant interaction terms, a simple slope analysis [54] was conducted by plotting psychological distress scores at low (−1 SD) and high (+1 SD) levels of loneliness or parenting anxiety at low and high levels of SNS use for parenting.

Statistical analyses were performed using JMP Pro 16.0 (SAS Institute) with a significance level of 5%. Sample size was calculated using G-power, assuming an effect size (*f*_2_) of 0.15 (medium), α error probability of .05, power of 0.8, and a number of predictors of 24, confirming that a sample size of 124 or more was required. In addition, in the analysis, when missing values for psychological distress, loneliness, parenting anxiety, and perceived social support were less than half of the items, missing values were imputed by predicting values based on the least squares method using the nonmissing parts of each scale. If more than half the items were missing, they were treated as missing data. Participants with missing values in nonpsychological scale items were also treated as having missing data. While participants with missing values were included in the analysis because of research ethics, they were excluded from the creation of multiple regression models. During this process, the usage of parenting-related SNSs and other psychological scale scores was compared between participants with missing data and those with no missing data, ensuring that there were no significant differences.

### Ethical Considerations

Informed consent and opt-out procedures were implemented as follows. First, individuals referred by the web survey company were provided with written information detailing the study's purpose, the voluntary nature of participation, the absence of any penalties for nonparticipation, the right to refuse to answer, the maintenance of anonymity, the use of data solely for research purposes, and the strict management of personal information. Second, participants were given the choice to either agree or decline participation in the survey; only those who clicked the consent button were considered to have consented to participate, thereby ensuring the opportunity to opt out. In addition, all survey questions included an option to decline to answer. Respondents’ personal information was not disclosed to the researchers, and the data remained fully anonymized, ensuring that researchers had no means of identifying individuals. Participants received digital reward points as compensation for their effort in completing the survey, in accordance with the web survey company’s guidelines. This study was conducted with the approval of the ethics committee for research involving human subjects at Toyama Prefectural University (approval number: R5-16, dated August 10, 2023).

## Results

### Descriptive Statistics

Among those who returned the survey, responses from 429 individuals with experience using SNSs for parenting and no missing data on SNS use were analyzed. Table 2 illustrates the descriptive statistics and differences between mothers (205 individuals; 205/429, 47.8%) and fathers (224 individuals; 224/429, 52.2%). The differences were related to parental age, age of the youngest child, employment status, educational level, and division of childcare and housework. No differences in psychosocial factors were determined between fathers and mothers.

**Table 2.** Descriptive statistics and gender differences (N=429).

			Mothers (n=205)				Fathers (n=224)					*t* test (*df*)	Chi-square (*df*)	*P* value
			n (%)		Mean (SD)		n (%)			Mean (SD)				
**Demographics**														
	Parent’s age (years)^a^		—^b^		34.5 (4.83)		—			39.8 (6.76)		8.85 (394)	—	<.001
	**Number of children**											1.72 (427)	—	.09
		1	94 (46)		—		91 (41)			—				
		2	87 (42)		—		92 (41)			—				
		≥3	24 (12)		—		41 (18)			—				
	**Youngest child’s age**											2.31 (427)	—	.02
		Infant	71 (35)		—		53 (24)			—				
		1 year	61 (30)		—		72 (32)			—				
		2 years	44 (21)		—		59 (26)			—				
		3 years	29 (14)		—		40 (18)			—				
	**Cohabiting family members**													
		Partner	196 (96)		—		220 (98)			—		—	2.47 (2)	.12
		Own family members	12 (6)		—		13 (6)			—		—	0.48 (2)	.79
		Partner’s family members	9 (4)		—		4 (2)			—		—	2.47 (2)	.12
	**Occupational status**											—	227.06 (3)	<.001
		Full-time worker	59 (29)		—		220 (98)			—				
		Part-time worker	23 (11)		—		1 (0)			—				
		Homemaker	76 (37)		—		1 (0)			—				
		On maternity or childcare leave	47 (23)		—		2 (1)			—				
	**Educational level (highest educational qualifications)** ^**c**^											—	33.91 (3)	<.001
		Junior high school/high school graduate	24 (12)		—		28 (13)			—				
		Junior college/vocational school graduate	63 (31)		—		23 (10)			—				
		University/graduate school graduate	117 (57)		—		172 (77)			—				
	**Subjective economic status**											–0.95 (427)	—	.34
		Very concerned	23 (11)		—		35 (15)			—				
		Somewhat concerned	90 (43)		—		89 (39)			—				
		Slightly concerned	82 (39)		—		97 (42)			—				
		Not concerned at all	14 (7)		—		9 (4)			—				
	Division of household and childcare responsibilities		—		74.8 (18.46)		—			34.4 (17.86)		–23.06 (427)	—	<.001
**Psychosocial factor**														
	Psychological distress^d^		—		2.12 (0.96)		—			2.09 (1.03)		–0.30 (423)	—	.77
	Loneliness^e^		—		2.31 (0.49)		—			2.35 (0.51)		0.80 (424)	—	.43
	Parenting anxiety^f^		—		2.44 (0.70)		—			2.37 (0.71)		–1.09 (424)	—	.28
	Perceived social support^g^		—		4.98 (1.48)		—			4.90 (1.29)		–0.62 (426)	—	.53

a Missing values: n=35 (mother: n=13; father: n=22).

b Not applicable.

c Missing values: n=2 (mother: n=1; father: n=1).

d Missing values: n=4 (mother: n=2; father: n=2).

e Missing values: n=5 (mother: n=1; father: n=2).

f Missing values: n=3 (mother: n=1; father: n=2).

g Missing value: n=1 (mother: n=1).

### Gender Differences in SNS Use

Table 3 presents the descriptive statistics and differences in SNS use between fathers and mothers. As regards digital device ownership, a significantly greater proportion of fathers owned a computer than mothers (*χ*²_1_=25.12; *P*<.001). In terms of SNS usage, a significantly higher percentage of mothers used Instagram than fathers (*χ*²_1_=41.87; *P*<.001). The frequency of SNS use showed that mothers were significantly more passive than fathers (*t*_427_=−2.45; *P*=.006).

**Table 3.** Gender differences in social networking sites use (N=429).

		Mothers (n=205)				Fathers (n=224)				*t* test (*df*)	Chi-square (*df*)	*P* value
		n (%)		Mean (SD)		n (%)		Mean (SD)				
**Having digital devices** ^a^												
	Having smartphones	203 (99)		—^b^		218 (97)		—		—	1.70 (2)	.19
	Having tablets	45 (22)		—		72 (32)		—		—	5.61 (2)	.02
	Having computers	86 (42)		—		148 (66)		—		—	25.12 (2)	<.001
**SNS^c^ use** ^**a**^												
	X (Twitter)	96 (47)		—		106 (47)		—		—	0.01 (2)	.92
	Instagram	147 (72)		—		91 (41)		—		—	41.87 (2)	<.001
	Facebook	65 (32)		—		79 (35)		—		—	0.61 (2)	.44
	Youtube	130 (63)		—		153 (68)		—		—	1.14 (2)	.29
	TikTok	30 (15)		—		29 (13)		—		—	0.26 (2)	.61
	Nonuse	26 (13)		—		35 (16)		—		—	0.76 (2)	.38
**Frequency of SNS use**												
	Active use	—		3.37 (2.5)		—		3.24 (2.49)		–0.53 (427)	—	.59
	Passive use	—		5.47 (2.4)		—		4.82 (2.45)		–2.78 (427)	—	.006
**SNSs for parenting**												
	Gathering parenting-related information	—		2.89 (1.25)		—		2.33 (1.25)		–4.62 (427)	—	<.001
	Gathering child-related information	—		2.79 (1.21)		—		2.29 (1.23)		–4.24 (427)	—	<.001
	Parenting insights browsing	—		2.49 (1.27)		—		2.18 (1.23)		–2.61 (427)	—	.01
	Observing into childhood moments	—		2.56 (1.25)		—		2.16 (1.24)		–3.33 (427)	—	.001
	Building a parenting community	—		2.02 (1.33)		—		1.96 (1.22)		–0.49 (427)	—	.63
	Communication among parenting peers	—		2.02 (1.33)		—		1.96 (1.22)		–0.27 (427)	—	.79
Total scale		—		2.46 (1.02)		—		2.15 (1.08)		–03.04 (427)	—	.003

a Multiple selection.

b Not applicable

c SNS: social networking site.

Regarding the frequency of SNS use for parenting, mothers were significantly more likely than fathers to gather parenting (*t*_427_=−4.62; *P*<.001) and child-related information (*t*_427_=−4.24; *P*<.001), browse parenting insights (*t*_427_=−2.61; *P*=.01), and observe childhood moments (*t*_427_=−3.33; *P*=.001). The mean scores for the frequency of SNS use for parenting, divided by the number of items, were 2.15 (SD 1.08) for fathers and 2.46 (SD 1.02) for mothers. An independent *t* test indicated that mothers had significantly higher scores than fathers (*t*_427_=−3.04; *P*=.003).

### The Effect of SNS Use for Parenting on Psychological Distress

Multiple regression models were created for both mothers and fathers to explain psychological distress with SNS use for parenting, loneliness, parenting anxiety, and covariates (Table 4). The model was constructed for mothers (*F*_12, 177_=11.78; *P*<.001; *R*²=0.44) and fathers (*F*_12, 189_=15.43; *P*<.001; *R*²=49.5). In the mothers model, a significant interaction effect was found only between parenting anxiety and SNS use for parenting (*b*=0.247, SE 0.091; *P*=.008). Meanwhile, in the fathers model, significant interaction effects were found between loneliness (*b*=0.324, SE 0.127; *P*=.01) and parenting anxiety (*b*=0.144, SE 0.069; *P*=.04) following SNS use for parenting.

**Table 4.** The effect of social networking sites use for parenting on psychological distress (N=392).

	Mothers (n=190)^a^						Fathers (n=202)^b^					
	*b* ^c^	SE	95% CL^d^		*β* ^e^	*P* value	*b*	SE	95% CL		*β*	*P* value
			LL^f^	UL^g^					LL	UL		
**Main effect**												
Loneliness	0.49	0.14	0.21	0.77	0.25	.001	0.40	0.16	0.07	0.72	0.20	.02
Parenting anxiety	0.51	0.09	0.33	0.68	0.37	<.001	0.42	0.10	0.23	0.60	0.29	<.001
SNS^h^ use for parenting	0.13	0.06	0.00	0.26	0.14	.04	0.23	0.07	0.09	0.36	0.24	.001
**Moderation effect**												
SNS use for parenting × loneliness	−0.17	0.12	−0.41	0.08	−0.09	.18	0.32	0.13	0.07	0.58	0.16	.01
SNS use for parenting × parenting anxiety	0.25	0.09	0.07	0.43	0.19	.008	0.14	0.07	0.01	0.28	0.13	.04
**Covariates**												
Parent’s age	−0.01	0.01	−0.03	0.02	−0.04	.54	0.00	0.01	−0.02	0.01	−0.02	.70
Number of children	−0.01	0.09	−0.18	0.16	−0.01	.89	0.07	0.08	−0.08	0.23	0.05	.33
Youngest child’s age	−0.04	0.06	−0.15	0.08	−0.04	.53	0.03	0.05	−0.07	0.14	0.03	.53
Subjective economic status	−0.16	0.07	−0.30	−0.01	−0.13	.03	−0.30	0.08	−0.45	−0.16	−0.23	<.001
Frequency of passive use of SNS	0.06	0.03	0.00	0.11	0.14	.03	0.00	0.03	−0.05	0.06	0.01	.90
Frequency of active use of SNS	−0.02	0.02	−0.07	0.03	−0.05	.42	0.00	0.03	−0.06	0.05	0.00	.96
Perceived social support	−0.05	0.05	−0.14	0.04	−0.08	.28	−0.15	0.06	−0.27	−0.04	−0.19	.009
Intercept	2.97	0.46	2.05	3.88	0.00	<.001	3.44	0.44	2.59	4.30	0.00	<.001

a Model fit: *F*=111.78; model *P* value <.001; *R*2=0.44; adjusted *R*2=0.41.

b Model fit: *F*=15.43; model *P* value <.001; *R*2=0.49; adjusted *R*2=0.46.

c *b*: estimate.

d CL: confidence limit.

e *β*: standardized estimate.

f LL: lower limit.

g UL: upper limit.

h SNS: social networking site.

### Moderation Effects of Loneliness and Parenting Anxiety

**Figure 2.**
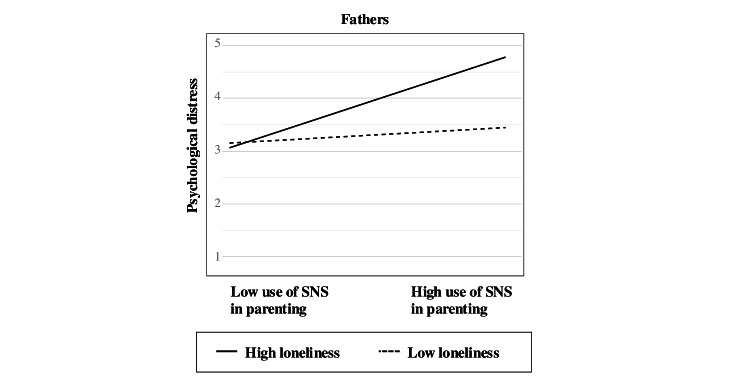
Simple slope analysis: interaction of parenting anxiety and use of SNSs in parenting predicting psychological distress. SNS: social networking site.

The results of the simple slope analysis are illustrated in Figures2  3. The mothers model showed that, at higher levels of parenting anxiety, SNS use for parenting was related to psychological distress (*b*=0.304, SE 0.090, *β*=.317; *P*<.001) but not at lower levels (*b*=−0.044, SE 0.090, *β*=−.046; *P*=.63). In the fathers model, SNS use for parenting was related to psychological distress at higher levels of parenting anxiety (*b*=0.330, SE 0.069, *β*=.346; *P*<.001) but not at lower levels (*b*=0.127, SE 0.097, *β*=.133; *P*=.19). In addition, SNS use for parenting was associated with psychological distress in fathers at higher levels of loneliness (*b*=0.390, SE 0.098, *β*=.409; *P*<.001) but not at lower levels (*b*=0.067, SE 0.089, *β*=.070; *P*=.45).

**Figure 3.**
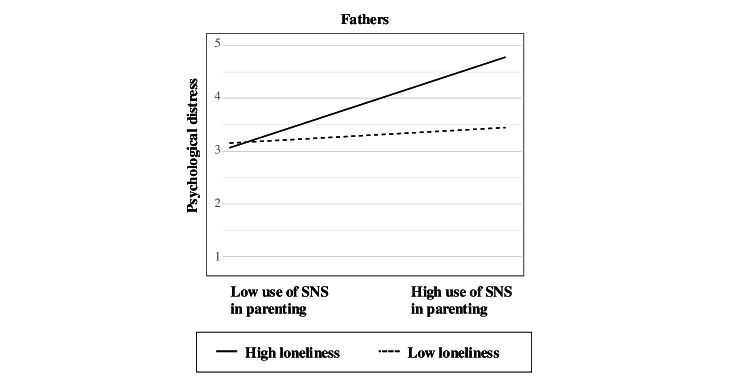
Simple slope analysis: interaction of loneliness and use of SNSs in parenting predicting psychological distress. SNS: social networking site.

## Discussion

### Major Findings

This study examined the moderating influence of loneliness and parenting anxiety on the relationship between SNS use for parenting and psychological distress among mothers and fathers of young children. We observed that mothers used parenting-related SNSs more frequently than fathers, primarily for gathering parenting information and monitoring others’ parenting activities. In addition, parenting anxiety moderated the relationship between SNS use for parenting and psychological distress in both genders, whereas the effect of loneliness was discernible only in fathers. Therefore, hypotheses 1 and 2 were fully supported and hypothesis 3 was partially supported. This novelty in our findings suggests the presence of both common and divergent elements in dynamics through which SNS use in parenting precipitates psychological distress in fathers and mothers. Previous research on SNS use and mental health has shown that the quality of interactions on SNSs (positive or negative) [55] and the attitude toward their use (passive or active) [56] can result in either positive or negative impacts on mental health. A systematic review of the relationship between SNS use, loneliness, and social anxiety [29] found that high levels of loneliness and social anxiety are correlated with problematic SNS use. However, it also highlighted the need for studies that simultaneously address these factors to explore their complex interrelationships. Most studies focusing on SNS use, loneliness, and anxiety have concentrated on adolescents, where SNS research is prevalent, leaving limited insights into both fathers and mothers in the context of early childhood parenting. This study extends previous findings by specifically examining SNS use in parenting and simultaneously addressing loneliness and anxiety to explore their moderating effects.

First, the moderating role of parenting anxiety was substantiated for both fathers and mothers regarding the association between SNS use in parenting and psychological distress. This suggests that parents struggling with heightened parenting anxiety may inadvertently exacerbate their psychological distress when using SNSs to cope with parenting challenges. An earlier study demonstrated that using information from smart devices to alleviate parenting concerns can paradoxically amplify anxiety when those concerns are severe [44]. Our findings align with previous research, hinting at the limited benefit for highly anxious parents in seeking comfort through SNSs. The background of psychological distress experienced by fathers and mothers with high parenting anxiety owing to SNS use may be related to low parenting self-efficacy and self-esteem, which are correlated with anxiety [27]. It is conceivable that parents burdened with acute parenting anxiety lack buffers such as self-efficacy and self-esteem against negative web-based influences, making them more vulnerable to psychological distress. In addition, parents prone to parenting anxiety inherently possess neurotic traits, which may foster upward social comparisons on SNSs and lead to increased psychological distress [57].

Second, the moderating effect of loneliness was observed solely among fathers, indicating that only those with significant levels of loneliness showed a strong correlation between intensive SNS use for parenting and high psychological distress. In general, higher levels of loneliness correlate with lower socioeconomic status [58]. In Japan, a persistent belief in gender roles assigns work responsibilities to men and domestic and child-rearing responsibilities to women [36]. Fathers of young children may identify strongly with the role of breadwinner and feel a deep sense of duty and pride. For fathers who feel isolated owing to socioeconomic constraints, SNS platforms that facilitate peer comparison [23] may exacerbate feelings of inadequacy regarding their family parenting practices, thus fostering psychological distress. Similarly, fathers who experience loneliness following strained partner relationships may face psychological distress from upward comparisons to seemingly idyllic families on SNSs. Furthermore, the lack of SNS platforms that naturally facilitate information sharing and camaraderie among fathers in the parenting domain may have also contributed to the distress experienced by lonely fathers. While social media and platforms for pregnant women and mothers are prevalent, there is a dearth of resources for fathers, including face-to-face peer support [39]. On more open platforms such as Instagram, X, and Facebook, fathers may find opportunities to connect with their peers, but in environments in Japan with strong gender-role divisions [36], they are likely to encounter more mother-centered information exchange. The use of SNSs in the context of fathers seeking connections and information sharing in parenting may have exacerbated psychological distress by creating a sense of alienation among them.

It is worth noting that no moderating effect of loneliness among mothers could be determined. This suggests that, regardless of loneliness, increasing SNS use for parenting among mothers is indicative of psychological distress. Previous studies have suggested that women experience significantly higher levels of depression owing to social comparisons on social media than men [59]. Mothers, as opposed to fathers, may be more susceptible to the mental health detriments of SNS use, regardless of whether or not they experience loneliness. In addition, the literature presents conflicting views on whether SNSs contribute positively by fostering desired social relationships [60,61] or negatively by forming unsatisfactory social connections for lonely individuals [18]. Previous research demonstrated that, for mothers in the parenting phase, the extent of their social networks on SNSs is inversely related to feelings of loneliness [62], suggesting that SNSs may not necessarily accentuate negative effects for lonely mothers.

Furthermore, given that this study is cross-sectional, there is the possibility of a reverse causal relationship, where parents with high levels of loneliness and parenting anxiety may engage more actively in SNS use for parenting because of their high psychological distress. Previous research reported that individuals with neurotic tendencies, which precede psychological distress, tend to use social media excessively or compulsively [63]. It is possible that parents experiencing high psychological distress, along with loneliness and parenting anxiety, may inherently possess neurotic tendencies and, consequently, may frequently use SNSs for parenting.

### Implications

This study highlights the differences in the moderating effects of loneliness and parenting anxiety on the relationship between SNS use and psychological distress among parents. It was observed that both fathers and mothers experiencing heightened levels of loneliness or parenting anxiety reported that SNS use in parenting activities was associated with increased psychological distress. This observation suggests that SNS use may not always be beneficial for parents experiencing loneliness or parenting anxiety, and it might be useful for professionals to discuss the potential drawbacks of relying heavily on SNSs for parenting support. Conversely, it is also suggested that parents experiencing high psychological distress, along with loneliness and parenting anxiety, may use SNSs frequently for parenting. As SNSs might serve as necessary support for parents in distress, it is crucial for professionals to support its moderate use while considering the limitations of its benefits. Given that SNS use for parenting is more common among mothers, it is important for professionals to recognize mothers as a group potentially more susceptible to both the positive and negative impacts of SNS use. Finally, enhancing SNS platforms specifically for lonely fathers as a parenting resource may be beneficial.

### Limitations

This study has several limitations. First, owing to its cross-sectional design, causality cannot be established. Second, the study relies on self-reported questionnaires, which does not eliminate the possibility of information bias. Third, the use of convenience sampling by a specific survey company in Japan may have introduced selection bias. Specifically, the sample may have been biased toward those with a high affinity for web-based information and high information literacy, thus limiting the generalizability of the results. Fourth, the study did not use a validated scale to measure SNS use for parenting, and although it considered real-life parenting situations through interview data, it lacks clear conceptualization and validation of construct validity, limiting the accuracy of the phenomenon measured. Fifth, the study did not consider the information literacy of the participants. Future research should use scales that more accurately measure the concept of SNS use in parenting. In addition, because a bidirectional causal relationship between SNS use and mental health is assumed, longitudinal studies that test for causality could provide higher-resolution insights into SNS use and parenting mental health.

### Conclusions

This study examined the moderating effects of loneliness and parenting anxiety on the relationship between SNS use and psychological distress among parents of young children. The moderating effect of parenting anxiety on this relationship was confirmed for both fathers and mothers, but the moderating effect of loneliness was established only for fathers. Given that different mechanisms underlie the loneliness experienced by fathers and mothers, the impact of SNS use on mental health may differ. Health and medical professionals might well need to tailor guidelines for SNS use in parental support taking into account gender. For fathers, a cautious approach to SNS use when feeling lonely is recommended, while for mothers, guidance on how to effectively balance SNS use with other support resources based on the level of parenting concerns may be needed.

## Supplementary material

10.2196/59029 Multimedia Appendix 1 Questionnaire.

## Abbreviations

SNS: social networking site
