# Supplementary material for: Parenting-Related Social Networking Site Use and Psychological Distress in Parents of Infants: Cross-sectional Study Exploring the Moderating Effects of Loneliness and Parenting Anxiety
Source: JMIR Pediatr Parent. 2024 Oct 11;7:e59029. doi: 10.2196/59029 (PMC11488460; doi:10.2196/59029)
Supplement: Multimedia Appendix 1 [file pediatrics-v7-e59029-s001.docx]

**Appendix 1.**

1. The survey items included in the online questionnaire administered in this study are presented below. Each question includes the answer option “do not wish to answer.”

Question: Your age

| ( ) years old |
| --- |

Question: Your sex

| 1. Male　2. Female　3. Other |
| --- |

Question: Number of children

| ( ) children |
| --- |

Question: Age of your youngest child

| ( ）Years ( ）Months |
| --- |

Question: Please select all other family members living with you besides your children

| 1. Spouse (including partner)  2. Your parents  3. Spouse's (including partner's) parents  4. Your siblings  5. Spouse's (including partner's) siblings  6. Others ( ) |
| --- |

Question: Your employment status

| 1. Full-time　2. Part-time　3. Homemaker　4. On maternity/paternity leave |
| --- |

Question: Highest level of education

| 1. Junior high school graduate　2. High school graduate  3. Vocational school graduate 4. Junior college graduate  5. University graduate　6. Graduate school graduate 6. Other ( ) |
| --- |

Question: Living situation

| 1. Very worried　2. Somewhat worried　3. Not so worried　4. Not worried at all |
| --- |

**2. We would like to ask about your use of social networking services (SNS). Please read what we mean by SNS in this survey before you answer.**

*In this survey, by SNS, we refer to online platforms where users can not only view information generated by their connections (friends and acquaintances) but also create their own content and respond to that information (e.g., comments, likes). Specifically, this includes platforms such as X (formerly Twitter), Instagram, Facebook, TikTok, YouTube, LinkedIn, etc. Please note that in this survey, LINE is not considered a part of SNS.*

**Question: Please select the digital devices you own.**

| 1. Smartphones 2. Tablets 3. Computers |
| --- |

**Question: Please select the social networking services (SNS) you use.**

| 1. X (Twitter) 2. Instagram 3. Facebook 4. YouTube  5. TikTok 6. Other Do not use any |
| --- |

**Question : Reflecting on the past week, please indicate your approximate frequency of SNS use.**

*Passive use: Scrolling through timelines, viewing/reading posts, watching videos, reading profiles, looking at pages liked by others, etc.*

*Active use: Posting photos, articles, or videos, liking or commenting on others' posts, sharing posts of interest, sending friend requests or follows, messaging, etc.*

|  | | **More than 10 times a day** | **More than 5 times a day** | **2**–**4 times a day** | **Once a day** | **3**–**6 times a week** | **1**–**2 times a week** | **Less than once a week** | **Not at all** |
| --- | --- | --- | --- | --- | --- | --- | --- | --- | --- |
| ① | **Passive use** | **8** | **7** | **6** | **5** | **4** | **3** | **2** | **1** |
| ② | **Active use** | **8** | **7** | **6** | **5** | **4** | **3** | **2** | **1** |

**3. We would like to ask about your use of SNS in parenting.**

**Question : How often do you use social networking services for parenting?**

| Item | | Never Use | Rarely Use | Sometimes Use | Often Use | Always Use |
| --- | --- | --- | --- | --- | --- | --- |
|  | Use to find out about parenting methods. | **1** | **2** | **3** | **4** | **5** |
|  | Use to find out about the child's growth and health. | **1** | **2** | **3** | **4** | **5** |
|  | Use to learn about parenting in other families’ children. | **1** | **2** | **3** | **4** | **5** |
|  | Use to learn about parenting in other families’ parenting. | **1** | **2** | **3** | **4** | **5** |
|  | Use to build parenting networks. | **1** | **2** | **3** | **4** | **5** |
|  | Use for communication with parenting peers. | **1** | **2** | **3** | **4** | **5** |

**5. We would like to ask about your parenting and mental state.**

**Question: Please select any number for the following statements that applies to you.**

| Item | | Do not think so | Do not think much | Somewhat think so | Think so |
| --- | --- | --- | --- | --- | --- |
|  | I get lost on what to do about childcare. | **1** | **2** | **3** | **4** |
|  | I worry that my child's development is delayed compared to other children. | **1** | **2** | **3** | **4** |
|  | I worry that my child is not growing as expected, fearing a delay in their development. | **1** | **2** | **3** | **4** |
|  | I worry that my child's development is delayed compared to what I see in parenting magazines, books, or online/app information. | **1** | **2** | **3** | **4** |
|  | Seeing what's on TV, in magazines/books, or online/apps makes me question whether my parenting is good enough. | **1** | **2** | **3** | **4** |
|  | I feel uncertain about my parenting compared to other mothers. | **1** | **2** | **3** | **4** |

**Question: Regarding the following statements, please indicate how often you typically feel this way.**

| Item | | Never | Almost never | Sometimes | Always |
| --- | --- | --- | --- | --- | --- |
|  | Do you ever feel like you lack social interactions with others? | **1** | **2** | **3** | **4** |
|  | Do you feel that you have a lot in common with the people around you? | **1** | **2** | **3** | **4** |
|  | Do you feel that you have people who are close to you in your life? | **1** | **2** | **3** | **4** |
|  | Do you ever feel left behind? | **1** | **2** | **3** | **4** |
|  | Do you ever feel like no one truly knows you well? | **1** | **2** | **3** | **4** |
|  | Do you ever feel isolated? | **1** | **2** | **3** | **4** |
|  | Do you feel that there is someone who truly understands you? | **1** | **2** | **3** | **4** |
|  | Do you ever feel unable to connect or experience a sense of unity with the people around you? | **1** | **2** | **3** | **4** |
|  | Do you feel that you have someone to talk to? | **1** | **2** | **3** | **4** |
|  | Do you feel that you have someone you can rely on? | **1** | **2** | **3** | **4** |

**Question: How often have you experienced the following in the past 30 days? Please check the box that applies.**

| Item | | Not at all | A little | Sometimes | Most of the time | Always |
| --- | --- | --- | --- | --- | --- | --- |
|  | Have you felt inexplicably exhausted? | **1** | **2** | **3** | **4** | **5** |
|  | Did you feel overly sensitive or on edge? | **1** | **2** | **3** | **4** | **5** |
|  | Did you feel so on edge that you couldn't settle down at all? | **1** | **2** | **3** | **4** | **5** |
|  | Did you feel a sense of despair? | **1** | **2** | **3** | **4** | **5** |
|  | Did you feel restless or uneasy? | **1** | **2** | **3** | **4** | **5** |
|  | Did you feel so restless that you couldn't sit still? | **1** | **2** | **3** | **4** | **5** |
|  | Did you feel gloomy? | **1** | **2** | **3** | **4** | **5** |
|  | Did you feel so down that nothing could cheer you up? | **1** | **2** | **3** | **4** | **5** |
|  | Did you feel that everything was a struggle? | **1** | **2** | **3** | **4** | **5** |
|  | Did you feel worthless? | **1** | **2** | **3** | **4** | **5** |

**Question: Please select any number for each of the following statements that applies to you.**

| Item | | Do not think so at all | Do not think so | Do not think much | Neutral | Somewhat think so | Think so | Very much think so |
| --- | --- | --- | --- | --- | --- | --- | --- | --- |
|  | I have someone who is there for me in times of trouble. | **1** | **2** | **3** | **4** | **5** | **6** | **7** |
|  | I have someone with whom I can share my joys and sorrows. | **1** | **2** | **3** | **4** | **5** | **6** | **7** |
|  | I have someone who can truly comfort me. | **1** | **2** | **3** | **4** | **5** | **6** | **7** |
|  | I have someone who is considerate of my feelings. | **1** | **2** | **3** | **4** | **5** | **6** | **7** |
